# Identification and validation of methylated *PENK* gene for early detection of bladder cancer using urine DNA

**DOI:** 10.1186/s12885-022-10275-2

**Published:** 2022-11-19

**Authors:** Tae Jeong Oh, Eunkyung Lim, Bo-Ram Bang, Justin Junguek Lee, Yong Gil Na, Ju Hyun Shin, Jae Sung Lim, Ki Hak Song, Sungwhan An

**Affiliations:** 1 Genomictree, Inc., 44-6 Techno 10-Ro Yuseong-Gu, Daejeon, 34027 Republic of Korea; 2 Promis Diagnostics Inc., 1 Post, Irvine, CA 92618 USA; 3 Department of Urology, Chungnam National University College of Medicine, 266 Munhwa-Ro Jung-Gu, Daejeon, 35015 Republic of Korea

**Keywords:** Bladder cancer, Methylation, Noninvasive, *PENK*, Urine sediment

## Abstract

**Background:**

Early detection of bladder cancer (BCa) offers patients a favorable outcome and avoids the need for cystectomy. Development of an accurate and sensitive noninvasive BCa diagnostic test is imperative. DNA methylation is an early epigenetic event in the development of BCa. Certain specific aberrant methylations could serve as useful biomarkers. The aim of this study was to identify methylation biomarkers for early detection of BCa.

**Methods:**

CpG methylation microarray analysis was conducted on primary tumors with varying stages (T1—T4) and paired nontumor tissues from nine BCa patients. Bisulfite-pyrosequencing was performed to confirm the methylation status of candidate genes in tissues and urine sediments (*n* = 51). Among them, *PENK* was selected as a potential candidate and validated using an independent set of 169 urine sediments (55 BCa, 25 benign urologic diseases, 8 other urologic cancers, and 81 healthy controls) with a quantitative methylation-specific real time PCR (me*PENK*-qMSP). All statistical analyses were performed using MedCalc software version 9.3.2.0.

**Results:**

CpG methylation microarray analysis and stepwise validation by bisulfite-pyrosequencing for tissues and urine sediments supported aberrant methylation sites of the *PENK* gene as potential biomarkers for early detection of BCa. Clinical validation of the me*PENK*-qMSP test using urine sediment-DNA showed a sensitivity of 86.5% (95% CI: 71.2 – 95.5%), a specificity of 92.5% (95% CI: 85.7 – 96.7%), and an area under ROC of 0.920 (95% CI: 0.863 – 0.959) in detecting Ta high-grade and advanced tumor stages (T1-T4) of BCa patients. Sensitivities for Ta low-grade, Ta high-grade, T1 and T2-T4 were 55.6, 83.3, 88.5, and 100%, respectively. Methylation status of *PENK* was not correlated with sex, age or stage, while it was associated with the tumor grade of BCa.

**Conclusions:**

In this study, we analyzed the comprehensive patterns of DNA methylation identified that *PENK* methylation possesses a high potential as a biomarker for urine-based early detection of BCa. Validation of *PENK* methylation confirms that it could significantly improve the noninvasive detection of BCa.

**Supplementary Information:**

The online version contains supplementary material available at 10.1186/s12885-022-10275-2.

## Background

Bladder cancer (BCa) is the 5th most commonly occurring cancer, with approximately 550,000 new cases and 200,000 deaths globally in 2018 [1, 2]. It is one of the cancers with the highest lifetime cost because it shows a high rate of recurrence and hence requires continuous invasive monitoring such as cystoscopy [3].

Cystoscopy is a gold standard for the diagnosis of primary tumor or recurrent urothelial carcinoma of the bladder. However, it is an invasive and costly procedure with low compliance [4, 5]. In addition, cystoscopy requires an experienced operator for thorough inspection and accurate diagnosis [6]. Although urine cytology is noninvasive and has high specificity for detection of BCa, its sensitivity is known to be as low as 20 to 50%, especially for low-grade tumors [7].

Numerous potential markers for detecting BCa have been described or are under investigation, and yet only a few have been validated to be clinically useful. The FDA has approved BTA STAT, BTA TRAK, NMP22/BladderChek, and UroVysion for diagnosis and follow-up while Immunocyt/uCyt is approved by the FDA only for follow-up [8, 9]. Among these, only UroVysion is a frequently used tool because it has been shown to be more sensitive than cytology. However, it is notable that the advantage of UroVysion over cytology in terms of sensitivity was largely lost when Ta samples from non-muscle invasive BCa were not included in the analysis [10, 11]. Individual markers have been shown to have insufficient diagnostic power presumably due to false-positive results, thereby decreasing the specificity of these markers. Therefore, the development of highly accurate and noninvasive methods using molecular biomarkers is crucial for early detection of BCa.

Epigenetic alterations are major mechanisms that can inactivate tumor suppressor genes and other cancer-associated genes in various cancers [12, 13]. Aberrant DNA methylation has been recognized as one of the most common molecular alterations in BCa [6, 14]. Detecting DNA hypermethylation in specific genomic regions of urine DNA has been shown to have high potential as a noninvasive diagnostic tool for early detection and surveillance of BCa [2, 15].

Various genome-wide strategies have been used to identify genes that undergo hypermethylation in BCa. Several urine-based epigenetic DNA markers have been shown to have potential for detecting BCa [14, 16–20]. In the present study, we conducted CpG microarray analysis to investigate differentially methylated sites of the genes in primary tumors and paired adjacent nontumor tissues of BCa. Step-wise validation procedures identified the methylation sites of the *PENK* gene as promising methylation biomarkers for the detection of BCa. Here, we report that *PENK* methylation assessment by methylation-specific real time PCR [21] can be used as a useful diagnostic tool for detection of BCa.

## Methods

### Clinical specimens

Fresh-frozen primary tumors and paired adjacent nontumor tissues from nine BCa patients (Stage T1, *n* = 5; T2, *n* = 1; T3, *n* = 1; T4, *n* = 2) were collected at the time of surgery. All frozen tissue specimens were obtained from the Chungnam National University Hospital. Each tumor specimen was histologically verified by a board-certified pathologist and archived for further DNA study. A total of 51 voided urine samples, used for bisulfite-pyrosequencing verification, were freshly obtained from BCa patients with varying stages (*n* = 16), patients with benign urologic diseases (BUD) such as trigonitis, urinary stone, and benign prostate hyperplasia (*n* = 23), and healthy individuals (*n* = 12). In the clinical validation for the urine DNA-based methylation test, an independent set of fresh voided urine samples were obtained from patients with BCa (*n* = 55) at various stages (Ta – T4), patients with BUD (*n* = 25), and normal healthy subjects (*n* = 81) as shown in Table 1. Additionally, urine samples from patients with kidney (*n* = 6) or prostate cancers (*n* = 2) were also included. All voided urine samples from BCa patients were collected before definitive surgery. Normal healthy control samples were obtained from individuals without any history of genitourinary malignancy. Voided urine samples (40 mL each) were collected into 50 mL tubes containing preservative buffer (Genomictree, Inc. Daejeon, South Korea), and were then centrifuged at 3000 × *g* for 10 min. The pelleted urine sediment was stored at -20 °C until DNA extraction. This study was approved by the Institutional Review Board of ChungNam National University Hospital, Daejeon, South Korea. Written informed consent was obtained from all study participants. This study adhered to local ethics guidelines.

**Table 1:** Clinicopathological features of subjects enrolled in this study

Characteristics	Tissues	Urine samples
**Healthy control**	-	93
Sex – no. (%)		
Male	-	60 (64.5)
Female	-	33 (35.5)
Age, mean (range)	-	53.8 (26–85)
**BUD**	-	48^a^
Sex – no. (%)		
Male	-	29 (60.4)
Female	-	19 (39.6)
Age, mean (range)	-	52.5 (34 – 83)
**Bladder cancer (BCa)**	9	71
Sex – no. (%)		
Male	7 (77.8)	55 (77.5)
Female	2 (22.2)	16 (22.5)
Age, mean (range)	74.3 (62—81)	68.8 (33 – 85)
Pathological stage – no. (%)		
Ta	-	28 (39.4)
T1	5 (55.6)	35 (49.3)
T2	1 (11.1)	4 (5.6)
T3	1 (11.1)	2 (2.8)
T4	2 (22.2)	2 (2.8)
Differentiation grade – no. (%)		
Low	4 (44.4)	35 (49.3)
High	5 (55.6)	33 (46.5)
Unknown	-	3 (4.2)

^a^ Benign urologic diseases (BUD) included trigonitis, urinary stone, and benign prostate hyperplasia

### CpG methylation microarray analysis

CpG methylation microarray analyses were performed using genomic DNA isolated from primary tumors and paired adjacent nontumor tissues from nine BCa patients with different stages (T1, *n* = 5; T2, *n* = 1; T3, *n* = 1; and T4, *n* = 2). CpG methylation microarray analysis was conducted as described previously [22] using human 244 k CpG island microarrays containing 237,000 oligonucleotide probes covering 27,800 annotated CpG islands (Agilent Technologies, CA, USA) according to the manufacturer’s instructions. Raw methylation microarray data were submitted to Gene Expression Omnibus (http://www.ncbi.nlm.nih.gov/geo) with accession number GSE171369.

Methylation microarray data were analyzed using the Agilent Feature Extraction software version 9.3.2.1 and a GeneSpring software version 7.3.1 (Agilent, CA, USA). To determine differentially methylated targets between primary tumor and paired adjacent nontumor tissue samples, statistical analysis was performed using a parametric analysis of variance test with Benjamini and Hochberg multiple testing correction (*P* < 0.01), followed by fold change analysis. Next, multiple-probe enriched genes were further selected as methylation candidate genes if their probes yielded a positive call for methylation in the bladder primary tumor compared to non-cancerous tissues with at least two probes.

### DNA extraction and bisulfite treatment

Genomic DNA from tissue specimens were extracted using a QIAamp DNA Mini kit (Qiagen, Hilden, Germany). Genomic DNA from urine sediments were isolated using a GT NUCLEIC ACID PREP kit (Genomictree, Inc., Daejeon, South Korea) according to the manufacturer’s instructions.

For methylation assessment, purified genomic DNA were first bisulfite-treated to convert unmethylated cytosine nucleotides into thymidine without changing methylated cytosines using an EZ DNA Methylation-Gold kit (Zymo Research, CA, USA) according to the manufacturer’s instructions. Briefly, DNA was chemically modified with sodium bisulfite at 64 °C in the dark for 2.5 h and then, the bisulfite-modified DNA was purified and eluted in 10 µL of distilled water. Eluted DNA was either immediately used for methylation analysis or stored at -20 °C until the analysis.

### Methylation assessment by bisulfite-pyrosequencing

To assess methylation status of candidate genes, bisulfite-pyrosequencing was performed as previously described [23]. Bisulfite PCR and pyrosequencing primers were designed to amplify 3 to 5 CpG dinucleotides sites in target regions of genes using a PSQ Assay Design software (Qiagen, Hilden, Germany). Sequences of primers used in pyrosequencing are listed in Table 2. These primers were synthesized by Bioneer (Daejeon, South Korea). Genomic DNA was modified by sodium bisulfite using an EZ DNA Methylation-Gold kit (Zymo Research, CA, USA) according to the manufacturer’s instructions. Briefly, 20 ng of bisulfite-treated DNA was amplified in a 25 µl reaction with primer set and Taq polymerase (Enzynomics, Daejeon, South Korea). PCR amplification was run for 40 cycles with an optimal annealing temperature.

**Table 2:** Primer sequences for pyrosequencing and me*PENK*-qMSP

Pyrosequencing		
**Gene**	**Sequences (5′ → 3’)**^**a**^	**Amplicon Size (bp)**
*CDX2*	F: TGGTGTTTGTGTTATTATTAATAG R: Biotin-CACCTCCTTCCCACTAAACTA S: ATTAATAGAGTTTTGTAAATAT	129
*CEI*	F: TGGAAATGTAAGTAGTTTTAGTGTAT R: Biotin-AAATTTCTTAACCAAACTTCTCATAT S: TGTAAGTAGTTTTAGTGTATTAAAT	152
*DMC1*	F: GAGGGGGGTAAGTGGTAAAAA R: Biotin-TCCCTCAAAATCACTAAA ATTCCT S: GGGGTAAGTGGTAAAAA	165
*IMP-1*	F: GGATTTYGAAAYGTTATTATTTAATAG R: Biotin-AACTAAAAACRAAATATCCCAAT S: ATTTYGAAAYGTTATTATTTAATAG	126
*PDE3A*	F: TGGGAATTTAGTGAAGAG R: Biotin-CCACTATAAACCAACTTATCCCTAACT S: GGGTATTTTATATTATGGTAGTG	84
*PENK*	F: ATATTTTATTGTATGGGTTTTTTAATAG R: Biotin-ACAACCTCAACAAAAAATC S: GGGTGTTTTAGGTAGTT	322
*SIM2*	F: Biotin-GTGGATTTAGATTAGGATTTTGT R: CACCCTCCCCAAATTCTT S: CCTCCCCAAATTCTTC	205
*VSX1*	F: GGAGTGGGATTGAGGAGATTT R: Biotin-AGTAAGTTTATGGGAGGGGGATT S: TTTTTGAAATGTTGGTAAG	89
*ZNF312*	F: AAGAGGGATTTGGAGAGAGAA R: Biotin-TCTCAATACACCCAACCTACATAC S: GATTTGGAGAGAGAAGG	140
**me*PENK*-qMSP**		
**Gene**	**Sequences (5′ → 3’)**^**a**^	**Concentration**
*COL2A1*	F: GTAATGTTAGGAGTATTTTGTGGGTA R: CTACCCCAAAAAAACCCAATCCTA P: Cy5-AGAAGAAGGGAGGGGTGTTAGGAGAGG	0.2 µM 0.2 µM 0.1 µM
*PENK*	F: TCGGGTGTTTTAGGTAGTTTCGC R: ACGACTCAAATCGCCTCGCG P: Fam-TGGGGGCGATCGCGTTATTTCGG	0.2 µM 0.2 µM 0.1 µM

^a^ F, R, S, and P represent forward, reverse PCR primers, sequencing primers, and PCR probe, respectively. Biotin, Cy5 or Fam indicates 5’ biotinylation, 5’Cy5 conjugation, and 5’Fam conjugation, respectively

Pyrosequencing was performed using a PyroGold kit and a PyroMarK ID Q96 instrument (Qiagen, Hilden, Germany) following the manufacturer’s instructions. Methylation index (MtI) of each gene in each sample was calculated as the average value of ^m^*C/*(^m^*C* + *C*) for all examined CpGs in target regions. All pyrosequencing reactions included samples without any DNA template as negative controls. Methylation-positive was considered if MtI of primary tumor was greater than that of the corresponding nontumor tissue.

### *PENK* methylation assessment in urine DNA by real time PCR

To measure *PENK* methylation quantitatively in DNA of urine sediment, total genomic DNA obtained from the sediments of voided urine was used for bisulfite treatment. All purified bisulfite-treated DNA was subsequently subjected to real time PCR-based methylation assessment for *PENK* (named as me*PENK*-qMSP). Primers and probes were designed to amplify the target region of *PENK* covering CpG targets (72 bp; + 524 to + 595 bp) and were synthesized by Integrated DNA Technologies (IDT) (IA, USA). me*PENK*-qMSP assay have been established with a modified protocol based on the report described by Eads et al*.* [21] in which the fluorescence-based qMSP quantitated the original methylation level of the interested gene locus using the bisulfite-converted sequences-specific primers and probes. Region of *COL2A1* DNA having no CpG sites was used for methylation-independent amplification as a control to determine the presence of bisulfite-treated DNA [24].

PCR reaction mixture contained 5 × AptaTaq PCR master mix (Roche Diagnostics, Mannheim, Germany), *PENK* methylation-specific primers and probes, and *COL2A1*-specific primers and probes (Table 2). me*PENK*-qMSP assay was performed on a 7500 Fast System Real-Time PCR (Thermo Fisher Scientific, MA, USA). Real time PCR was performed with the following thermal cycling conditions: activation at 95 °C for 5 min, 40 cycles of 95 °C for 15 s, and 60 °C for 1 min. Heating and cooling rates were set to ≥ 4 °C per sec and ≥ 3.5 °C per sec, respectively. For each experiment, BCa cell (RT4) genomic DNA containing fully methylated *PENK* gene and whole genome amplified genomic DNA of human lymphocyte containing unmethylated *PENK* gene [25] were used as controls to validate me*PENK*-qMSP adequacy of each sample batch. Non-template controls were also included for each experiment to detect cross-contamination. Cycle threshold (C_T_) values were analyzed using the 7500 software (Thermo Fisher Scientific, MA, USA). For urine DNA testing, C_T_ values for each experimental set were determined using a manually configured cutoff value. This cutoff value was established using the unmethylated DNA fluorescence level as a baseline. me*PENK*-qMSP was performed at one time for each sample. The relative level of methylated *PENK* gene in each sample was determined as 40-△C_T_ [C_T_ of amplified *PENK* gene – C_T_ of *COL2A1* (reference gene)] [26]. Higher values of 40-△C_T_ represented higher levels of *PENK* methylation. If C_T_ of *PENK* was undetected, the value was considered to be 25, the nearest value to the lowest of 40-△C_T_ for test results of all samples.

### Statistical analysis

All statistical analyses were performed using MedCalc software, version 9.3.2.0 (Basel, Belgium). A *P* value of less than 0.05 was considered statistically significant. Receiver operating characteristic (ROC), area under ROC (AUC), and 95% confidence intervals (CI) were calculated to confirm the accuracy of diagnosis, sensitivity, and specificity. Samples were categorized as negative or positive based on the cutoff value determined by the ROC curve analysis of the assay results.

## Results

### Identification and confirmation of methylation candidate genes for BCa detection

**Fig. 1:**
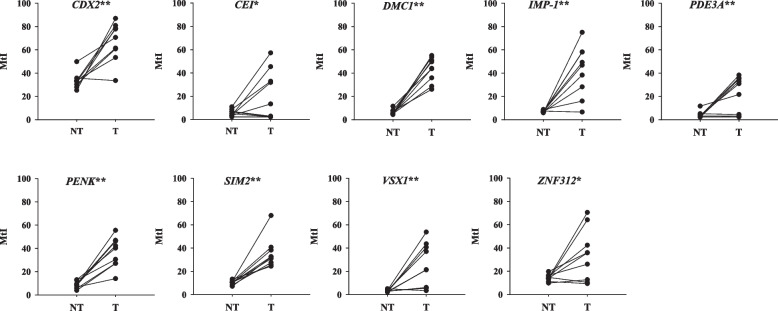
Assessment of methylation levels of nine candidates in bladder tissues by bisulfite-pyrosequencing. Methylation status was determined for nine genes in primary tumor and corresponding nontumor tissues used in CpG microarray analysis. MtI values are plotted from pyrosequencing results. Samples from the same patients are linked with a straight line. NT: Nontumor tissues, T: Tumor tissues. *, *P* < 0.05 and **, *P* < 0.01 analyzed by paired t-test

To identify a subset of candidate genes differentially hypermethylated in BCa, methylation profiles were compared between primary bladder tumors and paired adjacent nontumor tissues using CpG microarray analysis (Additional file 1: Figure S1). Statistical analysis and fold change analysis identified nine top-ranking hypermethylated candidate genes (*CDX2*, *CEI*, *DMC1*, *IMP-1*, *PDE3A*, *PENK*, *SIM2*, *VSX1*, and *ZNF312*) in primary bladder tumors. To confirm microarray results, bisulfite-pyrosequencing was subsequently performed for tissues used in microarray. Results revealed that all nine candidate genes were significantly hypermethylated in most primary tumors (*P* < 0.05) (Fig. 1). Among them, three genes (*DMC1*, *PENK*, and *SIM2*) were selected for further independent validation with additional normal tissues and BCa tissues because the genes showed higher methylation levels in all cancer tissues than in normal tissues. It was confirmed that methylation levels of three genes were significantly higher in most tumor tissues than in normal bladder tissues (*P* < 0.05, Kruskal Wallis test) (Additional file 2: Figure S2).

### Methylation status of *DMC1*, *PENK*, and *SIM2* genes in urine sediments by bisulfite-pyrosequencing

**Fig. 2:**
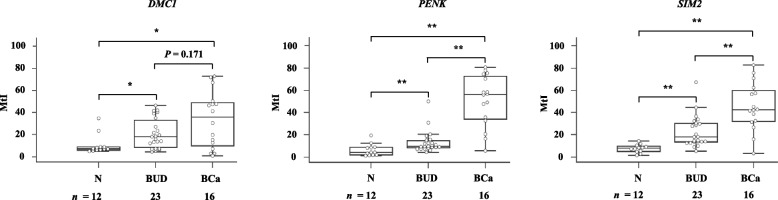
Methylation assessment of three genes in DNA from urine sediments by bisulfite-pyrosequencing. MtIs of samples are presented by box and whisker plots. Differences in MtI are statistically analyzed between BCa patients, BUD patients, and normal healthy subjects (N). *, *P* < 0.05 and **, *P* < 0.01 analyzed by Kruskal–Wallis test

To evaluate the possibility of using methylated genes *DMC1*, *PENK*, and *SIM2* with urine-based DNA test for clinical application, bisulfite-pyrosequencing was performed on urine sediment DNA from patients with BCa (*n* = 16), patients with BUD (*n* = 23), or healthy subjects (*n* = 12) (Fig. 2). The overall MtIs of genes *DMC1*, *PENK*, and *SIM2* in healthy controls were as low as 10.7 ± 9.0, 6.4 ± 5.4, and 7.5 ± 3.7, respectively. However, the overall MtIs of genes *DMC1*, *PENK*, and *SIM2* in BCa patients were significantly elevated at 33.6 ± 25.4, 51.0 ± 23.2, and 45.4 ± 21.6, respectively (*P* < 0.05). The overall MtIs of genes *DMC1*, *PENK*, and *SIM2* in urine samples from BUD patients were 20.9 ± 13.6, 13.3 ± 9.9, and 22.6 ± 14.2, respectively.

The overall MtIs of the *DMC1* gene in BCa patients were significantly higher than those in healthy individuals (*P* < 0.05), but not significantly higher than those in BUD patients (*P* = 0.171). The overall MtIs of *PENK* and *SIM2* genes were significantly higher across urine samples from BCa patients than those from patients with BUD or normal healthy subjects (*P* < 0.01) (Fig. 2). We focused on the *PENK* gene to pursue further clinical validation for urine-DNA test by methylation-specific real time PCR because it exhibited the best sensitivity and specificity for differentiating patients with BCa from control group such as patients with BUDs and healthy subjects (Additional file 3: Figure S3).

### Clinical validation of *PENK* methylation for detecting BCa in urine sediments by quantitative methylation-specific real time PCR

To assess *PENK* methylation precisely, we established and optimized *PENK* methylation-specific real time PCR, me*PENK*-qMSP assay and tested the sensitivity and specificity of *PENK* methylation with urine DNA from 55 patients with BCa at various stages (Ta – T4), 25 patients with BUD, and 81 healthy individuals.

**Fig. 3:**
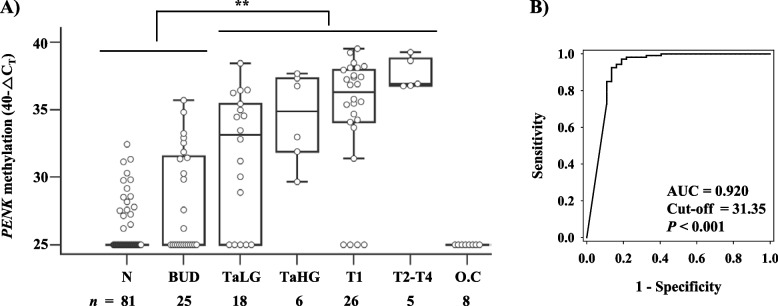
Methylation status of *PENK* in urine sediments by me*PENK*-qMSP. **A** me*PENK*-qMSP using an independent set of voided urine samples from BCa patients, BUD patients, and normal healthy subjects (N). Distribution of *PENK* methylation was expressed as 40-△C_T_ value for each sample. A higher 40-△C_T_ indicates a higher methylated level of *PENK*. Methylation status of *PENK* is plotted as box and whisker plots. TaLG: Ta Low-Grade; TaHG: Ta High-Grade; O.C.: Other urologic cancers. **, *P* < 0.01 analyzed by Kruskal–Wallis test. **B** ROC plots of *PENK* methylation for detecting TaHG and advanced tumor stages of BCa from BUD patients and normal healthy subjects. Cutoff value for methylation-positive, AUC, and *P* value are indicated in the box

Results of me*PENK*-qMSP analysis showed that levels of *PENK* methylation in urine DNA from BCa patients were significantly higher than in urine DNA from controls composed of patients with BUD and normal healthy subjects (*P* < 0.01, Kruskal–Wallis test) (Fig. 3A). Next, we evaluated the clinical performance of me*PENK*-qMSP for differentiating Ta high-grade and advanced tumor stages (T1-T4) of BCa from controls (patients with BUD and healthy normal subjects) by constructing an ROC curve. Given an optimal cutoff value at 31.35 of 40-△C_T_, the AUC was 0.920 (95% CI: 0.863 – 0.959, *P* < 0.001) and the overall sensitivity for detecting BCa at all stages (Ta through T4) was 76.4% (95% CI: 63.0 – 86.8%) with a specificity of 92.5% (95% CI: 85.7 – 96.7%). Sensitivities for Ta low-grade, Ta high-grade, T1 and T2-T4 were 55.6, 83.3, 88.5, and 100%, respectively. Sensitivity for detecting Ta high-grade and advanced stages of BCa patients was 86.5% (95% CI: 71.2 – 95.5%) (Fig. 3B). *PENK* methylation was not correlated with sex, age or stage (all *P* > 0.05, Fisher’s exact test). However, it was associated with tumor grade (*P* = 0.008, Fisher’s exact test) (Table 3). *PENK* methylation was not detected in all samples of other cancers including all renal cancer patients (*n* = 6) and prostate cancer patients (*n* = 2).

**Table 3:** The relationship between clinicopathological parameters and *PENK* methylation in urine sediments

Parameters	No. of total samples	No. of *PENK* methylation positive (%)
Sex		
Male	43	32 (74.4)
Female	12	10 (83.3)
*P* value^a^	0.709	
Age		
< 65	19	12 (63.2)
≥ 65	36	30 (83.3)
*P* value^a^	0.109	
Stage		
Ta, T1	50	37 (74.0)
T2 -T4	5	5 (100)
*P* value^a^	0.324	
Grade		
Low	26	16 (61.5)
High	28	26 (92.9)
*P* value^a^	0.008	

^a^ *P* value was calculated by Fisher’s exact test

## Discussion

Aberrant DNA methylation of some genes is known to be an early event in tumorigenesis. Specific methylation sites have been considered as potential biomarkers for early detection of cancer [12, 13, 27]. While several urine- marker tests such as NMP22, Immunocyt, BTA stat, and UroVysion have been approved by US FDA, most assays have not been proven to have sufficient sensitivity and/or specificity to be utilized in clinical practice [8]. Some other studies have reported that multiple genetic and epigenetic biomarkers [5, 28–30]-based tests have been evaluated for detecting BCa but they showed various range of sensitivities of 41.6 to 92.0% and specificities of 73.0 to 91.0%.

Here, we identified candidates of methylation biomarkers such as *DMC1*, *PENK*, and *SIM2* for BCa through comprehensive DNA methylation profiling analysis searching for differential methylation sites based on CpG microarrays and evaluation by pyrosequencing using clinical specimens. Based on a ROC curve and diagnostic model of BCa, we then selected *PENK* methylation as a biomarker for further clinical validation for the detection of BCa since *PENK* itself exhibited a highest sensitivity and specificity.

To assess the clinical performance of me*PENK*-qMSP for early detection of BCa, patients with varying stages of BCa (Ta – T4) were included in this study. The me*PENK*-qMSP test showed an overall sensitivity of 76.4% and a specificity of 92.5% in detecting BCa and the test had sensitivity of 86.5%, with high specificity of 92.5% when patients with Ta low-grade are not included in the analysis.

Since high-grade of T1 BCa and advanced stages are aggressive and have poor prognosis, early detection of high-grade T1 in BCa patients is crucial for decreasing morbidity and mortality [28, 31]. The me*PENK*-qMSP test in this study showed a good sensitivity of 89.5% (17/19) for high-grade T1 BCa while the sensitivity for low-grade Ta patients only was as low as 55.6% (10/18). The low sensitivity could be attributed to the cohesive nature of low-grade tumor cells, which may decrease the number of exfoliated cells in the urine [7].

In order to increase the sensitivity of the test, it is necessary also to improve the analytical sensitivity of the detection method to measure accurately trace amounts of *PENK* methylation in urine-DNA. Additionally, DNA integrity is greatly affected by the collection protocol and storage conditions of urine samples prior to downstream procedures for DNA methylation analysis [32]. Therefore, developing a better preservation buffer and optimizing urine collection procedures can enhance the assay sensitivity for methylated *PENK* DNA when detecting BCa in urine sediment.

We previously filed a patent application related to the detection of biomarkers for methylation in urine DNA such as *PENK* methylation which can be used for BCa diagnosis non-invasively. At that time, Chung et al. [26] had published clinical studies evaluating the clinical validity of *PENK* methylation in detection of BCa using urine sedimentation as *PENK* methylation had 81.3% sensitivity and 79.1% specificity for detection of BCa. In comparison to this study, our findings showed comparable sensitivity and higher specificity. Zhang et al. [33] also recently reported detecting BCa in urine samples by using *PENK* methylation combined with 6 additional methylation markers. In that study, however, the clinical performance of *PENK* methylation itself was not assessed. Despite this, taken together with previous studies indicate that *PENK* methylation has potential for the use of molecular biomarker in detection of BCa non-invasively.

Studies have shown that *PENK* hypermethylation is also associated with other cancers including hepatocellular carcinoma, colorectal cancer, and prostate cancer [34–36]. Therefore, we assessed whether *PENK* methylation was detectable in urine samples from other urologic cancers (two prostate cancer and six kidney cancer patients). However, we did not find aberrant *PENK* methylation in prostate cancer patients (0/2) or renal cancer patients (0/6). This indicates that *PENK* methylation in urine is highly specific for BCa.

The *PENK* gene encodes met-enkephalin (MENK), known as an opiate growth factor (OGF), has been reported in brain and prostate tumors. And it was previously reported as s a tonically active inhibitory factor that can interact with opioid growth factor receptors [37]. These reports may support, *PENK* as a tumor suppressor gene in several human tumors, including pancreatic cancer. In addition, MENK is required, in part, for apoptosis induction through transcriptional repression of NF-kB- and p53-regulated genes [38, 39].

This study has several limitations. First, patients with BCa (mean age: 69.1 years) were older than those with BUD and healthy subjects (mean age: 56.7 years) (*P* < 0.001, Kruskal–Wallis test). In addition, numbers of female BCa patients and other urological samples were small, leading to insufficient statistical power. In this study, *PENK* methylation was identified as a potential molecular biomarker for non-invasive diagnosis of bladder cancer. Because of limitations including the small number of samples, male-to-female ratio, and age-matching between BCa and non-BCa groups, it is not possible to draw any definitive conclusions at this time. Consequently, well-designed, large-scale clinical studies are required in order to determine whether this biomarker test using urine specimens is fully specific and accurate in detecting bladder cancer in clinical practice.

## Conclusions

We identified a specific aberrant *PENK* methylation in BCa through CpG microarray analysis and stepwise filtering procedures. This study showed that BCa can be detected noninvasively using a real-time PCR-based *PENK* methylation assay based on urinary DNA. However, a large scale prospective clinical trial utilizing the *PENK* methylation test for urine will need to be conducted before this method can be employed in clinical practice.

## Supplementary Information

**Additional file 1:** **Figure S1.** Stepwise filtering processes for candidate gene selection. Methylated DNA was separately enriched for DNA from nine primary bladder tumors and paired adjacent non-cancerous normal tissues with a MeDIA technique. Methylated DNA (Cy5) were individually compared with amplified common reference DNA (Cy3) without methylation enrichment. Statistically significant 2,887 hypermethylated probes were selected from 68,873 reliable probes. Then 277 CpG probes were further selected based on methylation mean fold-changes. Nine candidate genes hypermethylated in primary bladder tumors were finally selected.

**Additional file 2:** **Figure S2.** Assessment of methylation levels of three genes in bladder tissues by bisulfite pyrosequencing. Methylation status was examined for three genes in independent primary tumors (T) and normal tissues (N). Five normal bladder tissues were obtained from patients undergoing cystostomy surgery, bladder trauma repair surgery, or open cystolitholapaxy surgery and 10 primary bladder tumor tissues from stage I BCa patients were also obtained at the time of surgery. MtI values are plotted from pyrosequencing results. Gene names are indicated at the bottom. **, *P* < 0.01 analyzed by Kruskal-Wallis test.

**Additional file 3.** **Figure S3.** ROC plots of three genes for detecting BCa from BUD patients and healthy normal subjects. Cutoff value for methylation-positive, AUC, sensitivities, and specificities are indicated at the bottom. 

## Data Availability

Raw methylation microarray data were submitted to Gene Expression Omnibus (http://www.ncbi.nlm.nih.gov/geo) with accession number GSE171369.

## Abbreviations

BCa: Bladder cancer
BUD: Benign urologic disease
qMSP: Quantitative methylation-specific PCR
MeDIA: Methylated DNA Isolation Assay
MtI: Methylation index
*PENK*: Proenkephalin
ROC: Receiver operating characteristic
AUC: Area under ROC
