# Supplementary figures and images for: Identification and validation of methylated PENK gene for early detection of bladder cancer using urine DNA
Source: BMC Cancer. 2022 Nov 19;22:1195. doi: 10.1186/s12885-022-10275-2 (PMC9675278; doi:10.1186/s12885-022-10275-2)

**Figure S1.**

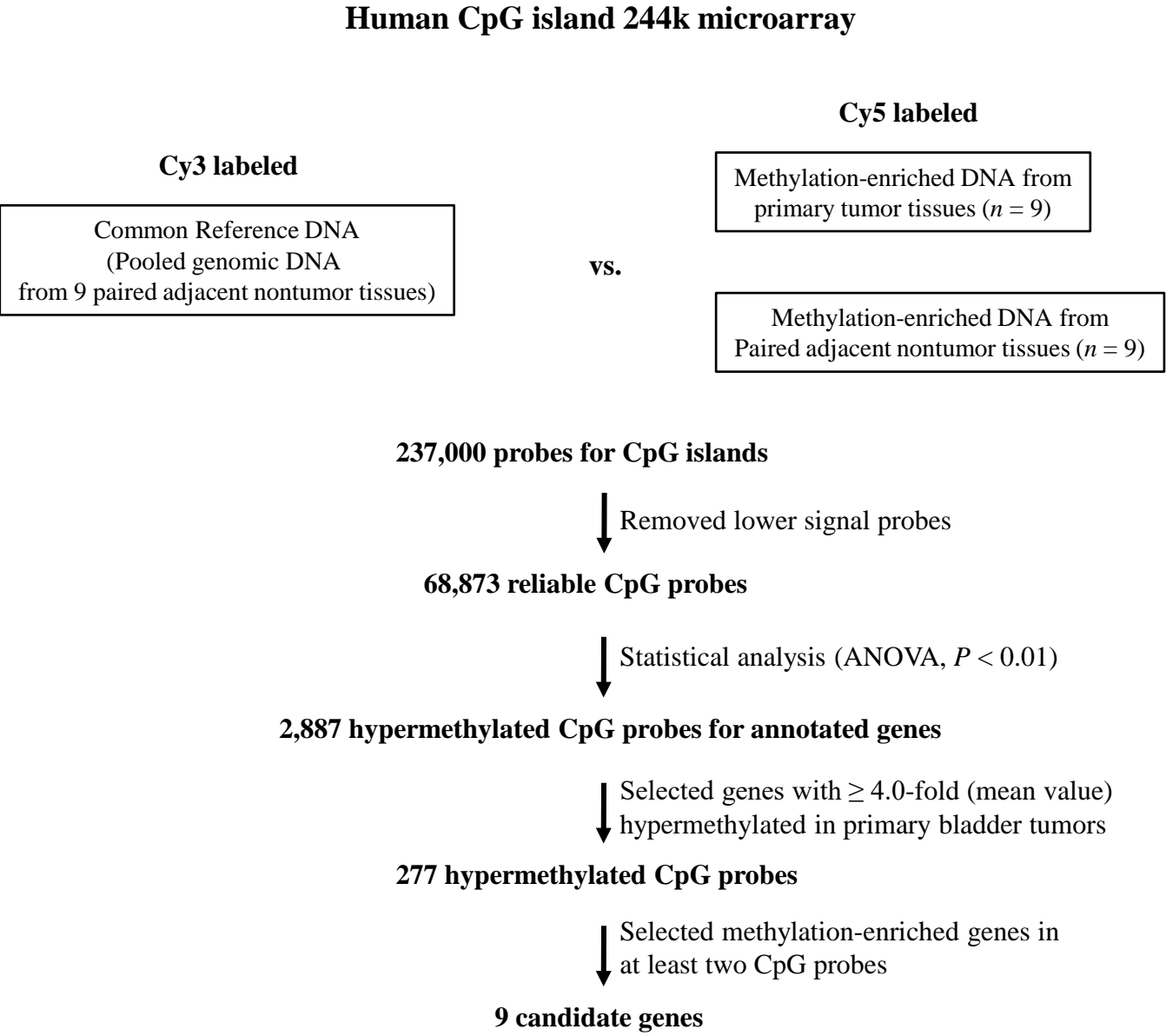

Supplement: Supplementary file 1 — Additional file 1: Figure S1. Stepwise filtering processes for candidate gene selection. Methylated DNA was separately enriched for DNA from nine primary bladder tumors and paired adjacent non-cancerous normal tissues with a MeDIA technique. Methylated DNA (Cy5) were individually compared with amplified common reference DNA (Cy3) without methylation enrichment. Statistically significant 2,887 hypermethylated probes were selected from 68,873 reliable probes. Then 277 CpG probes were further selected based on methylation mean fold-changes. Nine candidate genes hypermethylated in primary bladder tumors were finally selected. [file 12885_2022_10275_MOESM1_ESM.pdf]

Figure S2.

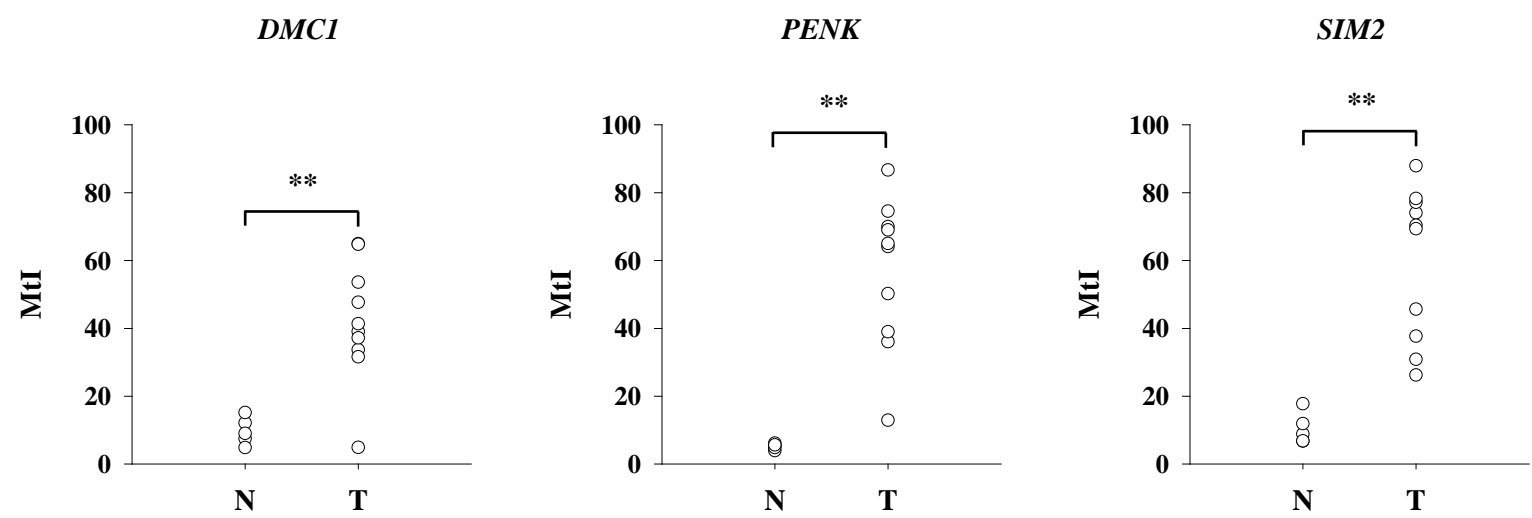

Supplement: Supplementary file 2 — Additional file 2: Figure S2. Assessment of methylation levels of three genes in bladder tissues by bisulfite pyrosequencing. Methylation status was examined for three genes in independent primary tumors (T) and normal tissues (N). Five normal bladder tissues were obtained from patients undergoing cystostomy surgery, bladder trauma repair surgery, or open cystolitholapaxy surgery and 10 primary bladder tumor tissues from stage I BCa patients were also obtained at the time of surgery. MtI values are plotted from pyrosequencing results. Gene names are indicated at the bottom. **, P < 0.01 analyzed by Kruskal-Wallis test. [file 12885_2022_10275_MOESM2_ESM.pdf]
