# Supplementary material for: Identification and validation of methylated PENK gene for early detection of bladder cancer using urine DNA
Source: BMC Cancer. 2022 Nov 19;22:1195. doi: 10.1186/s12885-022-10275-2 (PMC9675278; doi:10.1186/s12885-022-10275-2)
Supplement: Supplementary file 3 — Additional file 3. Figure S3. ROC plots of three genes for detecting BCa from BUD patients and healthy normal subjects. Cutoff value for methylation-positive, AUC, sensitivities, and specificities are indicated at the bottom. [file 12885_2022_10275_MOESM3_ESM.pdf]

Figure S3.

DMC1

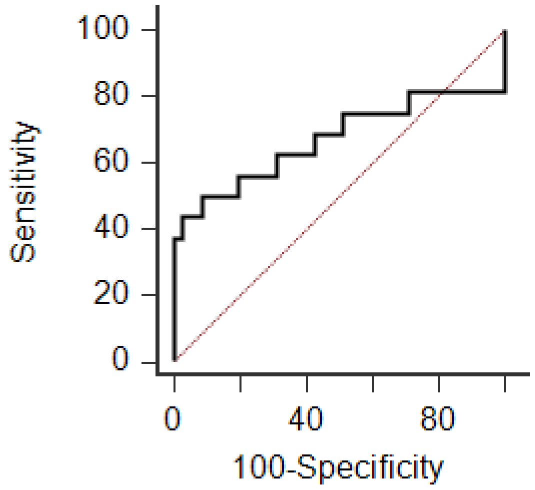

AUC = 0.670 (95% C.I: 0.524-0.795)
Cut-off 40.3
Sensitivity = 50.0% (95% C.I: 24.7-75.3%)
Specificity = 91.4% (95% C.I: 76.9-98.2%)

PENK

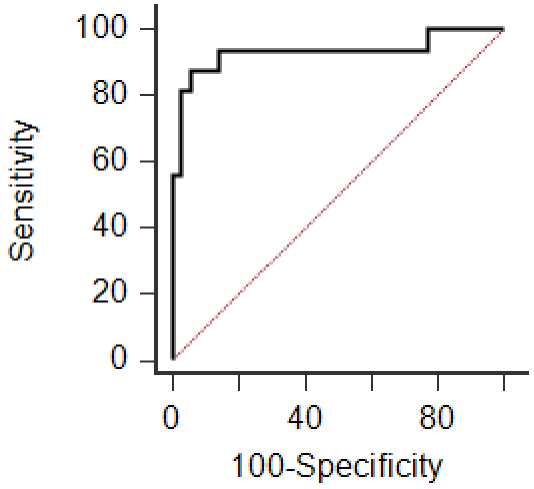

AUC = 0.932 (95% C.I: 0.741-0.945)
Cut-off 20.4
Sensitivity = 87.5% (95% C.I: 61.7-98.4%)
Specificity = 94.3% (95% C.I: 80.8-99.2%)

SIM2

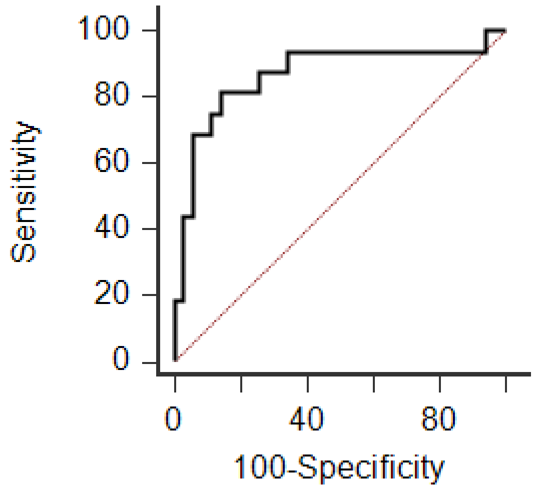

AUC = 0.866 (95% C.I: 0.741-0.945)
Cut-off 30.2
Sensitivity = 81.2% (95% C.I: 54.4-96.0%)
Specificity = 85.7% (95% C.I: 69.7-95.2%)
